# A randomized, double-blind, placebo-controlled study of high-dose bosentan in patients with stage IV metastatic melanoma receiving first-line dacarbazine chemotherapy

**DOI:** 10.1186/1476-4598-9-69

**Published:** 2010-03-30

**Authors:** Richard F Kefford, Philip R Clingan, Benjamin Brady, Andrea Ballmer, Adele Morganti, Peter Hersey

**Affiliations:** 1 Westmead Institute for Cancer research and Melanoma Institute of Australia, University of Sydney at Westmead Hospital, NSW 2145, Australia; 2 Medical Oncology, Southern Medical Day Care Centre, Crown St, Wollongong, NSW 2500, Australia; 3 Medical Oncology, Cabrini Hospital Malvern, Wattletree Rd, Malvern, Vic 3144, Australia; 4 Actelion Pharmaceuticals Australia Pty Ltd, Narabang Way, Belrose, NSW 2085, Australia; 5 Biometry Department, Actelion Pharmaceuticals, Via delle Valli, 26/18100, Imperia, Italy; 6 Newcastle Melanoma Unit, Calvary Mater Newcastle, Corner King & Platt Streets, Waratah, NSW 2300, Australia

## Abstract

**Background:**

The endothelin system is implicated in the pathogenesis of melanoma. We evaluated the effects of bosentan - a dual endothelin receptor antagonist - in patients receiving first-line dacarbazine therapy for stage IV metastatic cutaneous melanoma in a phase 2, proof-of-concept study.

**Results:**

Eligible patients had metastatic cutaneous melanoma naïve to chemotherapy or immunotherapy, no central nervous system involvement, and serum lactate dehydrogenase <1.5 × upper limit of normal. Treatment comprised bosentan 500 mg twice daily or matching placebo, in addition to dacarbazine 1000 mg/m^2 ^every three weeks. Eighty patients were randomized (double-blind) and 38 in each group received study treatment. Median time to tumor progression (primary endpoint) was not significantly different between the two groups (placebo, 2.8 months; bosentan, 1.6 months; bosentan/placebo hazard ratio, 1.144; 95% CI, 0.717-1.827; p = 0.5683). Incidences of most adverse events and clinically relevant increases in hepatic transaminases were similar between treatment groups although hemoglobin decrease to >8 and ≤ 10 g/dL and ≤ 8 g/dL was more common in the bosentan group.

**Conclusions:**

In patients receiving dacarbazine as first-line chemotherapy for metastatic melanoma, the addition of high-dose bosentan had no effect on time to tumor progression or other efficacy parameters. There were no unexpected safety findings.

**Trial registration:**

This study is registered in ClinicalTrials.gov under the unique identifier NCT01009177.

## Background

Each year there are approximately 160,000 new cases of malignant melanoma worldwide and it is responsible for an estimated 41,000 deaths [1]. The incidence is currently increasing and predicted to continue increasing for the next 20 years or more [2]; mortality is also increasing in many countries, including Europe and Australia [3]. The prognosis for patients with distant metastases from melanoma is poor; patients with elevated serum lactate dehydrogenase or extra-pulmonary visceral involvement have a median survival of just 4-6 months and a 2-year survival rate of ≤ 5% [4]. No systemic therapy has been shown in phase III trials to be superior to single agent treatment with dacarbazine. However, response rates of only 8-15% have been reported for this approach in randomized controlled trials [5-10]. Neither combination chemotherapy with tamoxifen [6] nor an intensive three-drug regimen with interferon-2-alpha and interleukin-2 showed any survival advantage over dacarbazine alone [11]. The addition of the anti-bcl2 oligonucleotide oblimersen showed no overall survival benefit over dacarbazine alone, except in a low lactate dehydrogenase (LDH) subgroup [9]. The dacarbazine-sensitizing drug lomeguatrib also showed no superiority in a double blind trial [12].

The endothelin (ET) pathway is involved in a number of aspects of melanocyte physiology and in the pathogenesis of melanoma [13-16]. The endothelin-B (ET_B_) receptor is expressed in primary and metastatic malignant melanoma, and increased expression of ET_B _correlates with tumor progression in malignant melanoma [15]. In addition, activation of the ET_B _receptor, by ET-1 and ET-3, results in downstream activation of tumor-promoting events and the progression of cutaneous melanoma [16].

The oral dual ET-receptor antagonist bosentan (Tracleer^®^, Actelion Pharmaceuticals Ltd.), indicated for the treatment of pulmonary arterial hypertension (PAH), blocks both ET_A _and ET_B _receptors. In human melanoma cell lines, bosentan has been observed to inhibit proliferation [13,17], decrease cell viability and DNA synthesis, and induce apoptosis [18]. Bosentan has also been shown to potentiate the effects of alkylating agents [18].

The results of a single-arm, phase II uncontrolled study indicated that bosentan monotherapy may be of benefit in patients with stage IV metastatic melanoma, achieving disease stabilization in six of 32 patients (18.8%) at week 6, with confirmation at week 12; five patients were still stable after 24 weeks and two remained stable after more than 2 years on study treatment [17]. We therefore hypothesized that ET receptor inhibition with bosentan may improve the efficacy of dacarbazine as therapy for metastatic melanoma, when the two agents are given in combination. The randomized, placebo-controlled, add-on phase 2, proof-of-concept, event-driven study presented here was conducted primarily to evaluate the effect of bosentan and placebo on time to tumor progression in patients with stage IV metastatic melanoma starting treatment with dacarbazine.

## Methods

### Patients

Patients eligible for the study were at least 18 years of age with histologically-proven malignant melanoma [4] and stage IV measurable disease as defined by Response Evaluation Criteria in Solid Tumors (RECIST) [19]. No prior therapy with dacarbazine was permitted and any prior radiation therapy was required to have been >30 days before study drug administration, with indicator lesions being either outside of the field of radiation or new, non-irradiated, lesions. Eastern-Cooperative Oncology Group (ECOG) performance status was required to be ≤ 2 and life expectancy >12 weeks. Female patients were required not to be pregnant or breastfeeding, and to be either post-menopausal, surgically sterile, or practicing a reliable method of contraception.

The main criteria for exclusion from the study were: lactate dehydrogenase >1.5 times the upper limit of normal (ULN); alanine aminotransferase (ALT) and/or aspartate aminotransferase (AST) >3 times the ULN at screening or ALT and/or AST >2 times the ULN and total bilirubin >2.0 mg/dL at screening; hemoglobin >30% below the lower limit of normal; central nervous system metastases or leptomeningeal metastases; any prior chemotherapy, biological therapy or immunotherapy for stage IV metastatic disease; immunotherapy <30 days before treatment start; ocular melanoma; known hypersensitivity to any excipients of bosentan; history of other malignancy in the past 5 years, with the exception of squamous cell carcinoma of the skin treated with local resection and basal cell carcinoma; planned use (or use within 4 weeks of the start of bosentan dosing) of another investigational drug; any standard contraindications to dacarbazine. Concurrent use of calcineurin inhibition, sirolimus, fluconazole or glyburide was not permitted. The study was conducted in full compliance with the principles of the Declaration of Helsinki and Good Clinical Practice. Local institutional review boards or independent ethics committees approved the protocol and all patients gave written informed consent.

### Treatment

Treatment with dacarbazine 1000 mg/m^2 ^every 3 weeks starting on Day 1 of the study was mandatory for all patients. In addition, eligible patients were randomized in a 1:1 double-blind manner to receive bosentan or matching placebo. Bosentan was administered orally with or without food at a dose of 500 mg twice a day (1000 mg total daily dose). This dose, equivalent to four times the standard daily bosentan dose used in the treatment of PAH, was selected on the basis of an earlier phase II study [17] and in vitro data from melanoma cell lines [13] in order to achieve maximum potential exposure. Four tablets (125 mg per tablet) were taken in the morning and four tablets approximately 12 hours later. Down-titration to 250 mg twice a day or 125 mg twice a day was allowed if the target study medication dose was not tolerated; however, it was recommended to maintain the full dose wherever possible.

### Evaluations

Tumor measurements including chest X-ray, computerized tomography scan, or magnetic resonance imaging were performed on Day 1, Week 6, every 6 weeks during dosing, and one week after the end of treatment. Assessment for tumor progression/response was performed at Week 6, every 6 weeks during dosing and one week after the end of treatment according to RECIST [19]. Patients underwent a series of laboratory tests including assessment of hematology (hemoglobin, hematocrit, erythrocyte count, leukocytes, platelets), liver function (ALT, AST, alkaline phosphatase, total and direct bilirubin) and chemistry (lactate dehydrogenase, sodium, potassium, blood urea nitrogen, creatinine) variables; these were performed at screening, Weeks 1-6, 8, 9, 12 and every 3 weeks thereafter during dosing, and at 1 and 4 weeks after the end of treatment. Assessments were scheduled for adverse events and serious adverse events on Day 1, at Weeks 1-6, 8, 9, 12 and every 3 weeks thereafter during dosing, and one week after the end of treatment. All new adverse events up to 24 hours after study drug discontinuation, and all new serious adverse events and grade 4/5 adverse events (National Cancer Institute Common Terminology Criteria for Adverse Events [NCI-CTCAE]) were reported up to 28 days after the end of treatment.

### Endpoints

The primary efficacy endpoint was time to tumor progression. Time to tumor progression was measured from randomization to the start of the first occurrence of one the following: objective or subjective (as per investigator's judgment) tumor progression; death due to disease progression; and initiation of other or additional anti-tumor therapy in the absence of documented tumor progression.

In the absence of an event as described above, patients were right censored at the last date of tumor assessment or at the randomization date in absence of post-randomization measurements. This rule was followed for patients on study treatment; patients being followed on study but off study treatment; patients lost to follow-up; patients withdrawn from the trial in the absence of progressive disease; and patients who died due to non-cancer-related causes. Secondary endpoints included progression-free survival and overall survival.

Safety endpoints included the incidence of treatment-emergent adverse events (NCI-CTCAE) occurring during and up to 1 day after end of study treatment; incidence of serious adverse events up to 28 days after end of study treatment; adverse events that led to discontinuation or dose reduction; incidences of worst CTCAE grade laboratory abnormalities during and up to 28 days after the end of study treatment; incidences of hemoglobin >8 and ≤ 10 g/dL, and ≤ 8 g/dL, with decrease from baseline; liver aminotransferases ≥ 3 times the ULN; total bilirubin ≥ 2 times the ULN with ALT and/or AST ≥ 3 times the ULN; increased blood alkaline phosphatase.

### Statistical methods

A total of 66 events were required at outset to detect a bosentan vs placebo hazard ratio (HR) of 0.5 with a type-I error of 0.05 (two-sided) and 80% power using the log-rank test to compare the distribution of time to tumor progression for the two treatment groups. Assuming enrolment of 70 patients (1:1 randomization) over 70 weeks, 66 events were expected to occur within 105 weeks if the median time to tumor progression was 10 weeks in the placebo group and the HR was 0.5. Efficacy data were analyzed on the all-randomized population comprising all randomized patients, whether or not they received any study drug. Safety data were analyzed on the all-treated population comprising all randomized patients who received study drug.

## Results

### Baseline demographics

**Figure 1:**
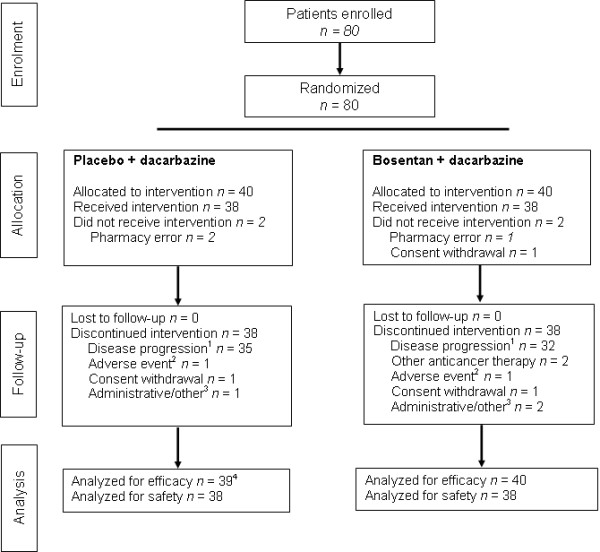
**Patient flow through the study**. Patient flow through the study: ^1^Including adverse events relating to disease progression; ^2^Unrelated to disease progression; ^3^Includes one patient in each group who discontinued due to study closure; ^4^Efficacy was analyzed on the all-randomized set; however, one patient, randomized to placebo, did not receive any study treatment, had no post-baseline tumor assessments, and had data missing for the date of randomization at the time of database closure. Because time-to-event analyses required a randomization date, this patient could not be included in these analyses, including the primary endpoint.

The study was performed in 11 centers in Australia, with the first patient, first visit in September 2005 and the last patient, last visit in February 2008. A total of 80 patients were randomized; however, two patients in each group did not receive any study treatment due to withdrawal of consent or randomization error by the pharmacy (Figure 1), and were excluded from the safety evaluation.

Baseline demographics are summarized in Table 1. Compared with the placebo group, the bosentan group comprised a higher proportion of males and the mean age of patients was slightly higher. The median number of target and non-target lesions was five (min, 2; max, 9) in the placebo group and five (min, 1; max, 10) in the bosentan group, the most common sites being the lung (placebo, 81.6%; bosentan, 59.0%), lymph nodes (31.6%; 38.5%), adrenal glands (5.3%; 28.2%), soft tissue (21.1%; 25.6%) and liver (18.4%; 23.1%). The percent of patients with lactate dehydrogenase levels ≤ ULN and >ULN was similar between the two treatment groups, and mean and median levels were comparable.

**Table 1:** Baseline demographics and clinical characteristics

Parameter	Placebo + dacarbazine (n = 40)*	Bosentan + dacarbazine (n = 40)*
Sex		
Male, n (%)	22 (55.0)	28 (70.0)
Female, n (%)	18 (45.0)	12 (30.0)
Mean age ± SD, years	58.0 ± 14.8**	62.1 ± 12.2
ECOG performance status, n (%)		
0	29 (72.5)	27 (67.5)
1	7 (17.5)	11 (27.5)
2	4 (10.0)	2 (5.0)

Lactate dehydrogenase	**Placebo +** **dacarbazine** **(n = 38)^†^**	**Bosentan +** **dacarbazine** **(n = 38)^†^**

≤ ULN, n (%)	25 (67.5)^‡^	26 (38.4)
> ULN, n (%)	12 (37.4)^‡^	12 (31.5)
Mean ± SD, U/L	351 ± 199^‡^	337 ± 178
Median [min, max], U/L	279 [99, 813]^‡^	259 [129, 774]

**Previous medications for stage I-III melanoma**	**Placebo +** **dacarbazine** **(n = 38)^†^**	**Bosentan +** **dacarbazine** **(n = 38) †**

Any previous treatment, n (%)	9 (23.7)	9 (23.7)
Immunostimulants	4 (10.5)	7 (18.4)
Interferons	4 (10.5)	1 (2.6)
Anti-neoplastic agents	1 (2.6)	1 (2.6)
Other investigational drug	1 (2.6)	1 (2.6)

SD, standard deviation of the mean; ECOG, Eastern Cooperative Oncology Group; ULN, upper limit of normal (value set by each local site, as per their normal practice)

* All-randomized set

** n = 39 (one patient missing randomization date; calculation of age not possible)

† All-treated population; data not available for 2 patients in each group (due to consent withdrawal or randomization error)

^‡ ^n = 37 (one patient missing)

### Exposure

In each group, 38 patients were treated and evaluable for exposure. For dacarbazine, the mean (± standard deviation) number of cycles administered was 5.5 (± 4.5) in the placebo plus dacarbazine treatment group and 4.8 (± 4.1) in the bosentan plus dacarbazine treatment group. Median duration of dacarbazine exposure (min; max) was 12.0 weeks (3.0; 61.0) and 7.5 weeks (3.0; 51.1) in the two treatment groups, respectively. The median daily exposure for bosentan (min; max) was 1000 mg (125 mg; 1000 mg) and the median duration of exposure (min; max) was 8.1 weeks (3.0; 71.0).

### Efficacy

**Figure 2:**
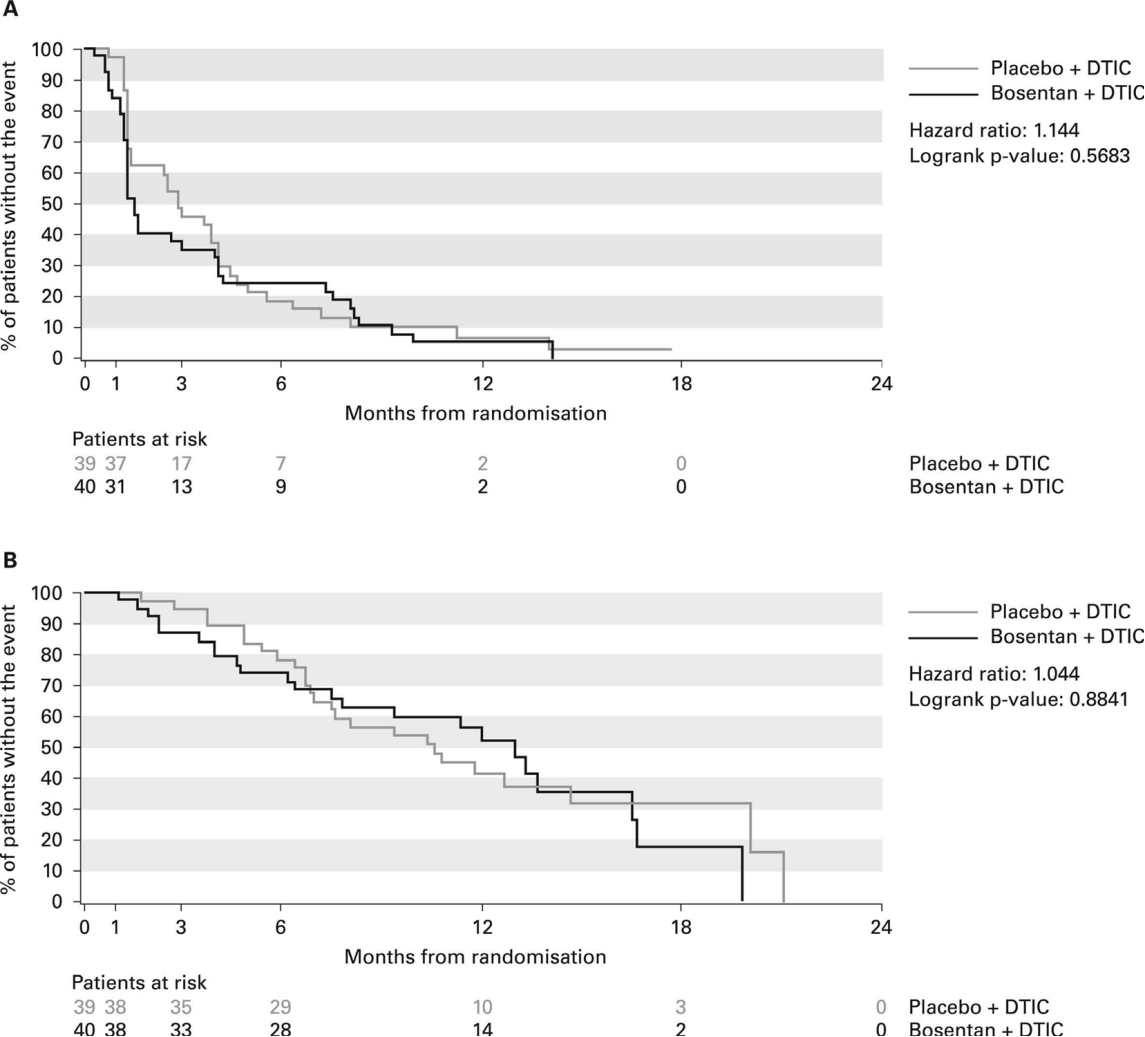
**Primary and secondary efficacy endpoints: Kaplan-Meier estimates of time to progression and overall survival**. Kaplan-Meier estimates of A) time to progression and B) overall survival (all-randomized population).

The primary endpoint, time to tumor progression, was not significantly different between the treatment groups, with a median of 2.8 months (95% CI, 1.4-4.1) for placebo and 1.6 months (95% CI, 1.3-4.0) for bosentan (HR, 1.144; 95% CI, 0.717-1.827; p = 0.5683) (Figure 2a). Supportive analysis on the all treated and per-protocol sets showed similar results (data not shown).

The secondary endpoints of median overall survival (Figure 2b) and progression-free survival were also not significantly different between treatment groups. Median overall survival was 10.6 months (95% CI, 6.9-14.7) with placebo and 13.0 months (7.8-16.6) with bosentan (HR, 1.044; 95% CI, 0.584-1.865; p = 0.8841) (Figure 2b). The Kaplan-Meier estimate for overall survival at 12 months was 42.1% (95% CI, 25.8-58.4) and 52.1% (95% CI, 35.1-69.1) for the two treatment groups, respectively. Median progression-free survival was 2.8 months (95% CI, 1.4-4.1) for placebo and 1.6 months (95% CI, 1.3-4.1) for bosentan (HR, 1.064; 95% CI, 0.664-1.704; p = 0.7944). The Kaplan-Meier estimate for progression-free survival at 6 months was 18.9% (95% CI, 6.3-31.6) and 27.0% (95% CI, 12.7-41.3) for the two treatment groups, respectively.

### Safety and tolerability

Adverse events occurring during and up to 1 day after the end of study treatment are shown in Table 2. All but one patient experienced an adverse event and similar proportions of patients in the two groups experienced a serious adverse event (placebo plus dacarbazine, 31.6%; bosentan plus dacarbazine, 34.2%). Anemia, thrombocytopenia, vomiting and lethargy were more common among patients receiving bosentan than placebo (all NCI CTCAE grades; ≥ 10% total difference). Conversely, fatigue, headache, dyspnea and upper respiratory tract infection were more common among patients receiving placebo.

**Table 2:** Incidence of NCI-CTCAEs up to one day post study treatment (all-treated population)

	Placebo + dacarbazine (n = 38)			Bosentan + dacarbazine (n = 38)		
	
Event	Any grade	Grade 3	Grade 4	Any grade	Grade 3	Grade 4
Patients with at least one AE, n (%)	37 (97.4)	9 (23.7)	1 (2.6)	38 (100)**	14 (36.8)	1 (2.6)
Patients with AE,* n (%)						
Nausea	19 (50.0)	-	-	22 (57.9)	1 (2.6)	-
Fatigue	19 (50.0)	-	-	14 (36.8)	2 (5.3)	-
Constipation	10 (26.3)	-	-	12 (31.6)	-	-
Vomiting	8 (21.1)	-	-	12 (31.6)	2 (5.3)	-
Thrombocytopenia	4 (10.5)	1 (2.6)	-	9 (23.7)	2 (5.3)	1 (2.6)
Headache	11 (28.9)	1 (2.6)	-	7 (18.4)	-	-
Anemia	1 (2.6)	-	-	7 (18.4)	2 (5.3)	1 (2.6)
Anorexia	7 (18.4)	-	-	6 (15.8)	-	-
Diarrhea	7 (18.4)	-	-	6 (15.8)	-	-
Neutropenia	5 (13.2)	2 (5.3)	1 (2.6)	6 (15.8)	5 (13.2)	-
Lethargy	2 (5.3)	-	-	6 (15.8)	-	-
Dyspnea	9 (23.7)	3 (7.9)	-	5 (13.2)	2 (5.3)	-
Cough	6 (15.8)	-	-	5 (13.2)	-	-
Pain in extremity	4 (10.5)	1 (2.6)	-	5 (13.2)	1 (2.6)	-
Edema peripheral	3 (7.9)	-	-	5 (13.2)†	-	-
Insomnia	5 (13.2)	-	-	4 (10.5)	-	-
Alanine aminotransferase increased	3 (7.9)	-	-	4 (10.5)	1 (2.6)	-

AE, adverse event

* ≥ 10% frequency in bosentan + dacarbazine group; **one patient had a Grade 5 event (metastatic malignant melanoma/malignant neoplasm progression); †data missing for one patient

Hematology assessment was graded according to the NCI CTCAE system and is reported in Table 3 as incidences of worst grades experienced during and up to 28 days after the end of study treatment. Grade 4 events occurred in two patients: one patient (2.6%) in the placebo plus dacarbazine group had Grade 4 reduction in neutrophils and one patient (2.6%) in the bosentan plus dacarbazine group had Grade 4 reduction in hemoglobin. Grade 3 hematology abnormalities occurred in no more than two patients (5.3%) in either treatment arm for any variable. Grades 2, 3, and 4 reductions in hemoglobin occurred with a higher incidence in the bosentan plus dacarbazine treatment group than in the placebo plus dacarbazine treatment group.

**Table 3:** Incidence of worst NCI-CTCAE hematology grades during and up to 28 days after the end of study treatment (all-treated population)

	Placebo + dacarbazine (n = 38)				Bosentan + dacarbazine (n = 38)			
	
	Grade 1	Grade 2	Grade 3	Grade 4	Grade 1	Grade 2	Grade 3	Grade 4
Hemoglobin, n (%)	26 (68.4)	2 (5.3)	-	-	19 (50.0)	9 (23.7)	1 (2.6)	1 (2.6)
Leukocytes, n (%)	8 (21.1)	6 (15.8)	2 (5.3)	-	10 (26.3)	7 (18.4)	1 (2.6)	-
Neutrophils, n (%)	4 (10.5)	5 (13.2)	2 (5.3)	1 (2.6)	3 (7.9)	8 (21.1)	1 (2.6)	-
Platelets, n (%)	11 (28.9)	3 (7.9)	-	-	10 (26.3)	3 (7.9)	2 (5.3)	-

Clinically significant laboratory abnormalities were also documented. Four patients (10.5%) in each treatment group had liver aminotransferases ≥ 3 times the ULN; 3 (7.9%) in each group had liver aminotransferases ≥ 3 and <5 times the ULN; and 1 (2.6%) in each group had liver aminotransferases ≥ 5 and <8 times the ULN. No patient in either treatment group experienced aminotransferase elevations ≥ 8 times the ULN. Reductions in hemoglobin, with decreases from baseline, occurred more often in the group receiving bosentan plus dacarbazine than the group receiving placebo plus dacarbazine: hemoglobin decreased to >8 and ≤ 10 g/dL in 10 patients (26.3%) and to ≤ 8 g/dL in 3 patients (7.9%) with bosentan plus dacarbazine; the corresponding changes occurred in only one patient (2.6%) each with placebo plus dacarbazine. No patients experienced bilirubin ≥ 2 times the ULN.

There were three deaths among patients receiving bosentan (all attributed to disease progression, with additional ascites in one case and hyponatremia in another case) and two deaths among patients receiving placebo (one due to disease progression and one due to cerebrovascular accident). All of the five deaths occurred after study treatment was stopped (during the 28-day follow-up) and none were considered, by the investigators, to be related to study treatment.

Adverse events were responsible for premature treatment discontinuation in two patients in the placebo plus dacarbazine group and three patients in the bosentan plus dacarbazine treatment group. In the placebo plus dacarbazine group, one of the two patients had an adverse event denoting disease progression and the other had an increase in serum ALT and alkaline phosphatase levels that was considered to be related to treatment and led to discontinuation of both placebo and dacarbazine. In the bosentan plus dacarbazine group, two of the three patients had adverse events denoting disease progression and the other had anorexia, fatigue and nausea, with the fatigue and nausea considered to be related to both bosentan and dacarbazine.

## Discussion

In this multicenter, randomized, double-blind, placebo-controlled, phase 2, proof-of-concept study, the addition of high-dose bosentan to treatment in patients receiving first-line dacarbazine for stage IV metastatic melanoma had no effect on time to tumor progression, overall survival or progression-free survival. For the primary endpoint of time to tumor progression, the median duration was 2.8 months among patients who received placebo and dacarbazine and 1.6 months among those who received bosentan and dacarbazine.

Previous studies with dacarbazine in metastatic melanoma have demonstrated median time to tumor progression of 2.3 to 2.7 months [5,8], progression-free survival of 1.5 to 2.7 months [5,7,9] and overall survival of 6.3 to 10.4 months [5-9]. The median overall survival observed in the placebo arm of the present study (10.6 months) is at the top of the previously reported range for dacarbazine monotherapy. The median overall survival in the bosentan arm (13.0 months) is also relatively high for a combination treatment in this setting [5-9]. A meta-analysis of 42 phase II trials in metastatic melanoma suggested that an agent in phase II testing should reach a 6-month progression-free survival of approximately 25% and a 12-month overall survival of approximately 45% to be considered for further evaluation in phase III [20]. In this study, PFS at 6 months was 18.9% with placebo and 27.0% with bosentan and overall survival at 12 months was 42.1% with placebo and 52.1% with bosentan. These figures lie in the upper quartile of values found in that meta-analysis and this may be partly attributed to the stringent selection criteria of no central nervous system metastases and low serum LDH.

This proof-of-concept study was designed to demonstrate a 50% risk reduction in favor of bosentan in time to tumor progression. Though this may seem ambitious, a hazard ratio of 0.5 in an event-driven trial of a drug given at high dose is not unrealistic in a phase 2, proof-of-concept study.

Bosentan has been extensively studied in patients with PAH in both randomized clinical trials and post-marketing surveillance [21-24]. This study in patients with melanoma used a dose of bosentan four times higher than the standard dose in PAH. Nevertheless, treatment was generally well tolerated in both the bosentan and placebo groups, and only five patients in total discontinued from the study due to adverse events. There were no deaths during the study treatment period and none considered to be related to treatment. Similarly, there were no unexpected safety concerns in the previous phase II study of bosentan monotherapy in metastatic melanoma [17], which used the same bosentan dose (500 mg twice daily) as the present study. Elevation of hepatic aminotransferases is an ET receptor antagonist class effect and is associated with their chronic use in PAH [24-26]. The incidence of clinically relevant increases in liver aminotransferases (>3 times the ULN) in our study in patients with melanoma is within the range reported for PAH patients receiving bosentan 125 mg bid [23,24,27].

In the present study, bosentan and placebo were administered in combination with first-line dacarbazine. The safety profile of dacarbazine is well established in the treatment of metastatic melanoma. Adverse events known to be associated with dacarbazine include myelosuppression (thrombocytopenia, anemia, and neutropenia), nausea and vomiting [reviewed in [28]]. Adverse events reported in the placebo arm of the present study were broadly in line with those reported for dacarbazine, although only one case (2.6%) of NCI-CTCAE anemia was reported in the placebo plus dacarbazine group, which is lower than might be expected based on historical data.

A decision was made when planning the study not to restrict accrual to patients with readily accessible tumor for fresh biopsy; subsequently, few tumor biopsy samples were obtained. This restricted ET receptor analysis as it can only be assayed in fresh frozen material by reverse-transcriptase polymerase chain reaction and is not amenable to immunohistochemical evaluation. Hence, it is not possible to comment on whether ET receptor expression was related to response to treatment. In the previous phase II study, a limited number of assays were performed; no correlation between ET receptor expression and response was observed [17].

## Conclusion

The addition of high-dose bosentan had no effect on time to tumor progression or other efficacy parameters in patients receiving dacarbazine as first-line systemic therapy for metastatic melanoma. The results of our study further highlight the challenges of treating metastatic melanoma and, in particular, improving outcomes beyond the modest benefits achieved with dacarbazine monotherapy.

## Competing interests

RK received travel assistance from Actelion Pharmaceuticals Ltd to present the trial at international meetings.

PRC has no conflict of interest. He participates in pharmaceutical company funded drug trials but receives no money for any of the trials. He has no shares in pharmaceutical companies. He sits on the advisory board for Eli Lilly regarding research in lung cancer.

BB has no conflicts of interest.

AB and AM and are employees of Actelion Pharmaceuticals Ltd, the manufacturers of bosentan.

PH has no conflicts of interest.

## Authors' contributions

RK contributed to the study design, recruited patients, was involved in data collection and data analysis and had a major role in writing the paper.

PRC was involved in patient recruitment.

BB was involved in patient recruitment.

AB contributed to the study design, was involved in the study conduct from start up to close out and contributed to the writing of the paper.

AM contributed to study design, was involved in data analysis.

PH was involved in patient recruitment.

All authors read and approved the final manuscript.
